# The genome sequence of the Brown Oak Tortrix, *Archips crataeganus *(Hübner, 1796)

**DOI:** 10.12688/wellcomeopenres.20413.1

**Published:** 2024-01-08

**Authors:** Douglas Boyes, Melanie Gibbs, University of Oxford and Wytham Woods Genome Acquisition Lab, Darwin Tree of Life Barcoding collective, Wellcome Sanger Institute Tree of Life programme, Wellcome Sanger Institute Scientific Operations: Sequencing Operations collective, Tree of Life Core Informatics collective, Darwin Tree of Life Consortium

**Affiliations:** 1 UK Centre for Ecology & Hydrology, Wallingford, England, UK

**Keywords:** Archips crataeganus, Brown Oak Tortrix, genome sequence, chromosomal, Lepidoptera

## Abstract

We present a genome assembly from an individual female *Archips crataeganus* (the Brown Oak Tortrix; Arthropoda; Insecta; Lepidoptera; Tortricidae). The genome sequence is 626.9 megabases in span. Most of the assembly is scaffolded into 31 chromosomal pseudomolecules, including the Z and W sex chromosomes. The mitochondrial genome has also been assembled and is 16.64 kilobases in length. Gene annotation of this assembly on Ensembl identified 19,596 protein coding genes.

## Species taxonomy

Eukaryota; Metazoa; Eumetazoa; Bilateria; Protostomia; Ecdysozoa; Panarthropoda; Arthropoda; Mandibulata; Pancrustacea; Hexapoda; Insecta; Dicondylia; Pterygota; Neoptera; Endopterygota; Amphiesmenoptera; Lepidoptera; Glossata; Neolepidoptera; Heteroneura; Ditrysia; Apoditrysia; Tortricoidea; Tortricidae; Tortricinae; Archipini; *Archips; Archips crataeganus* (Hübner, 1796) (NCBI:txid1857967).

## Background

The Brown Oak Tortrix, *Archips crataeganus* (Hübner, 1796), from the family Tortricidae is found in Europe, Asia Minor and north-western Africa. The East Asian (South Korea, Japan, China: Heilongjiang, Jilin, Shaanxi, Sichuan) subspecies, *A. crataegana endoi*, was described by Yasuda (1975). In the UK, *A. crataegana* is classified as ‘local’; uncommon but with a wide distribution over much of the British Isles ( Davis, 2012) *.* In the UK, *Archips crataegana* is mainly found in wooded habitats and has one generation per year. Adults fly between June and August. This species is sexually dimorphic ( Szabóky & Csóka, 2010); females are larger than males. Males have light brown forewings with dark brown markings, females tend to have darker forewings with obscured markings ( Bradley *et al.*, 1973). Females deposit egg masses on the bark of a variety of deciduous trees, including *Quercus*, *Betula*, *Fraxinus* and *Salix* species ( Szabóky & Csóka, 2010). The egg masses resemble bird droppings, and the eggs overwinter ( Szabóky & Csóka, 2010). The larvae (particularly the later instars) feed inside tightly rolled leaves ( Szabóky & Csóka, 2010). Pupation occurs at the final larval feeding site. Like other members of the genus *Archips*, the larvae of this species are polyphagous pests of fruit and forest trees, causing damage to leaves, blossoming buds, flowering buds and flowers ( Meijerman & Ulenberg, 2000).

The genome of the Brown Oak Tortrix, *Archips crataeganus*, was sequenced as part of the Darwin Tree of Life Project, a collaborative effort to sequence all named eukaryotic species in the Atlantic Archipelago of Britain and Ireland. Here we present a chromosomally complete genome sequence for *Archips crataeganus*, based on one female specimen from Wytham Woods, Oxfordshire, UK.

## Genome sequence report

**Figure 1.**
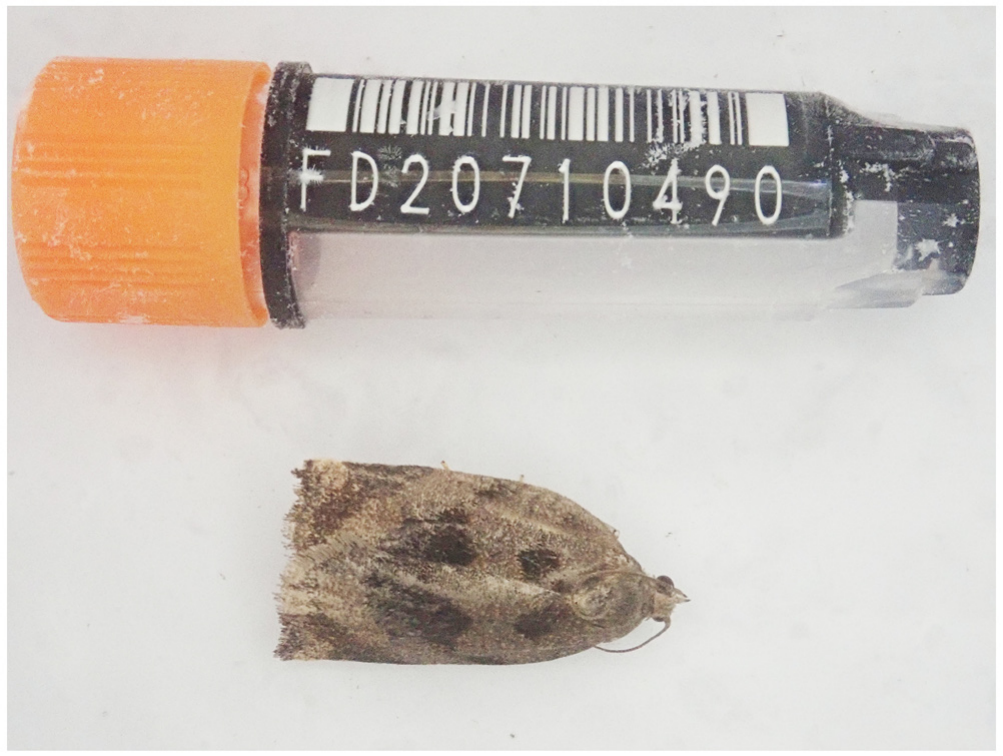
Photograph of the *Archips crataeganus* (ilArcCraa1) specimen used for genome sequencing.

The genome was sequenced from one female *Archips crataeganus* ( Figure 1) collected from Wytham Woods, Oxfordshire (biological vice-county Berkshire), UK (51.77, –1.34). A total of 36-fold coverage in Pacific Biosciences single-molecule HiFi long reads was generated. Primary assembly contigs were scaffolded with chromosome conformation Hi-C data. Manual assembly curation corrected 38 missing joins or mis-joins and removed 7 haplotypic duplications, reducing the scaffold number by 50%, and increasing the scaffold N50 by 3.1%.

**Figure 2.**
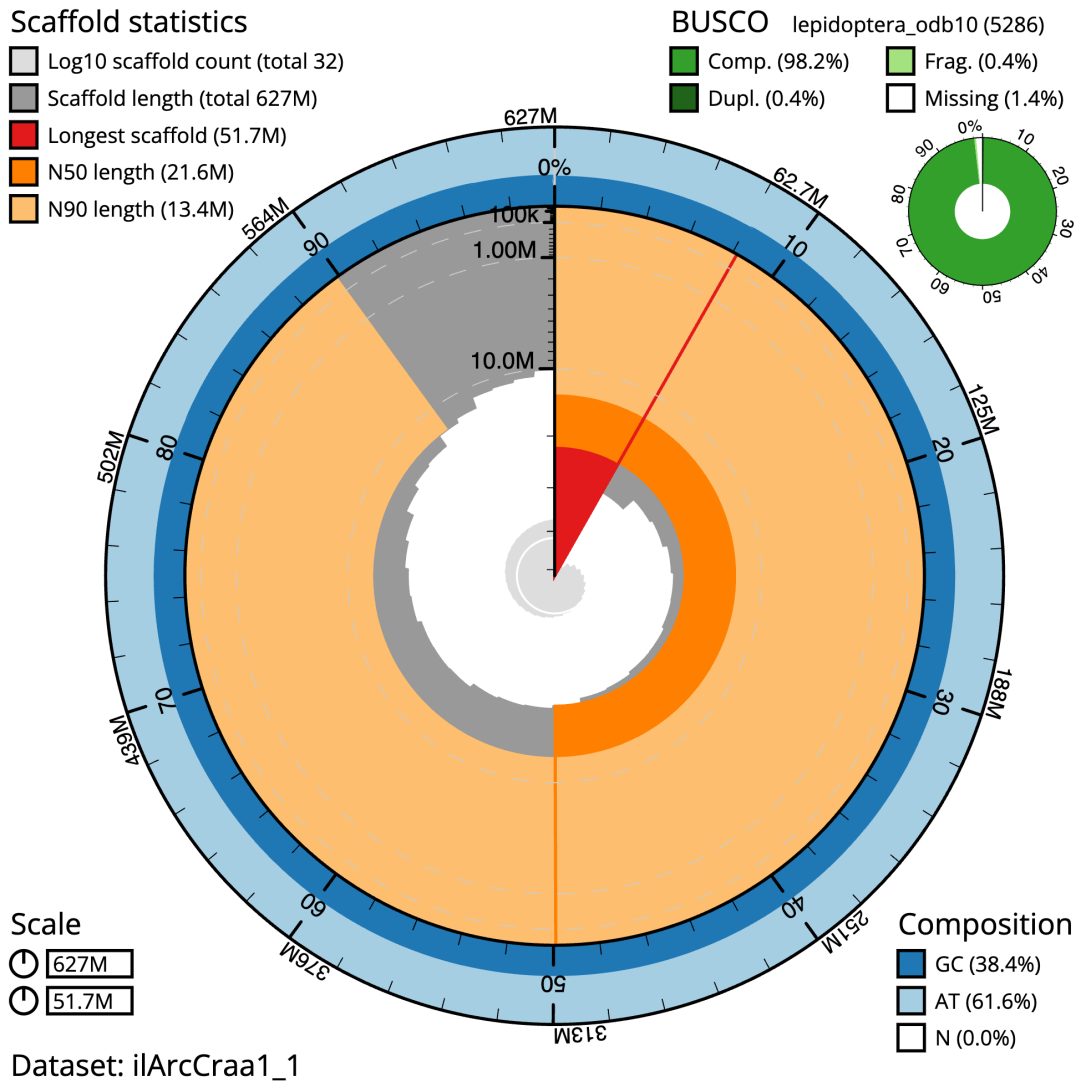
Genome assembly of *Archips crataeganus*, ilArcCraa1.1: metrics. The BlobToolKit Snailplot shows N50 metrics and BUSCO gene completeness. The main plot is divided into 1,000 size-ordered bins around the circumference with each bin representing 0.1% of the 626,923,680 bp assembly. The distribution of scaffold lengths is shown in dark grey with the plot radius scaled to the longest scaffold present in the assembly (51,685,801 bp, shown in red). Orange and pale-orange arcs show the N50 and N90 scaffold lengths (21,612,927 and 13,401,462 bp), respectively. The pale grey spiral shows the cumulative scaffold count on a log scale with white scale lines showing successive orders of magnitude. The blue and pale-blue area around the outside of the plot shows the distribution of GC, AT and N percentages in the same bins as the inner plot. A summary of complete, fragmented, duplicated and missing BUSCO genes in the lepidoptera_odb10 set is shown in the top right. An interactive version of this figure is available at https://blobtoolkit.genomehubs.org/view/Archips%20crataeganus/dataset/ilArcCraa1_1/snail.

**Figure 3.**
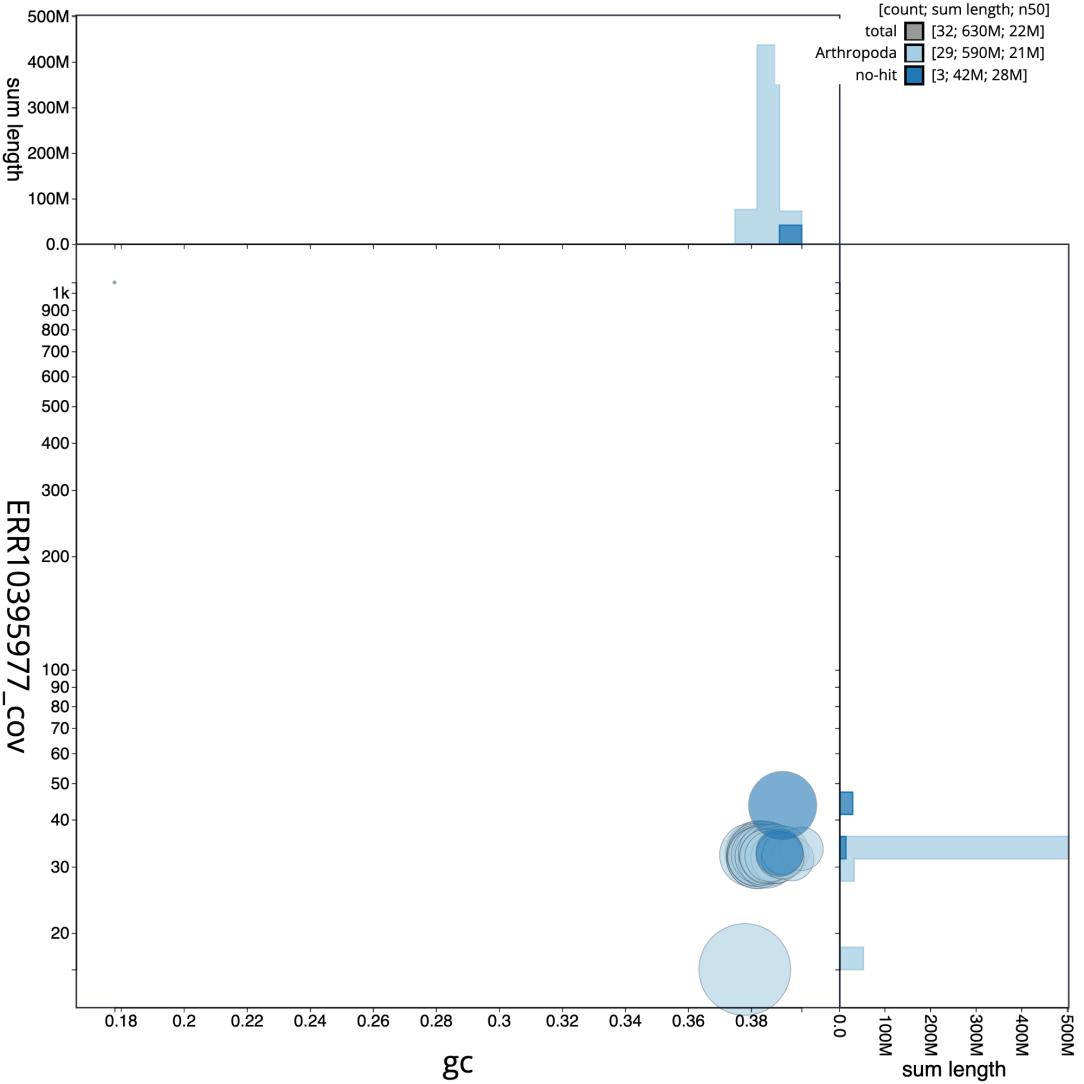
Genome assembly of *Archips crataeganus*, ilArcCraa1.1: BlobToolKit GC-coverage plot. Scaffolds are coloured by phylum. Circles are sized in proportion to scaffold length. Histograms show the distribution of scaffold length sum along each axis. An interactive version of this figure is available at https://blobtoolkit.genomehubs.org/view/Archips%20crataeganus/dataset/ilArcCraa1_1/blob.

**Figure 4.**
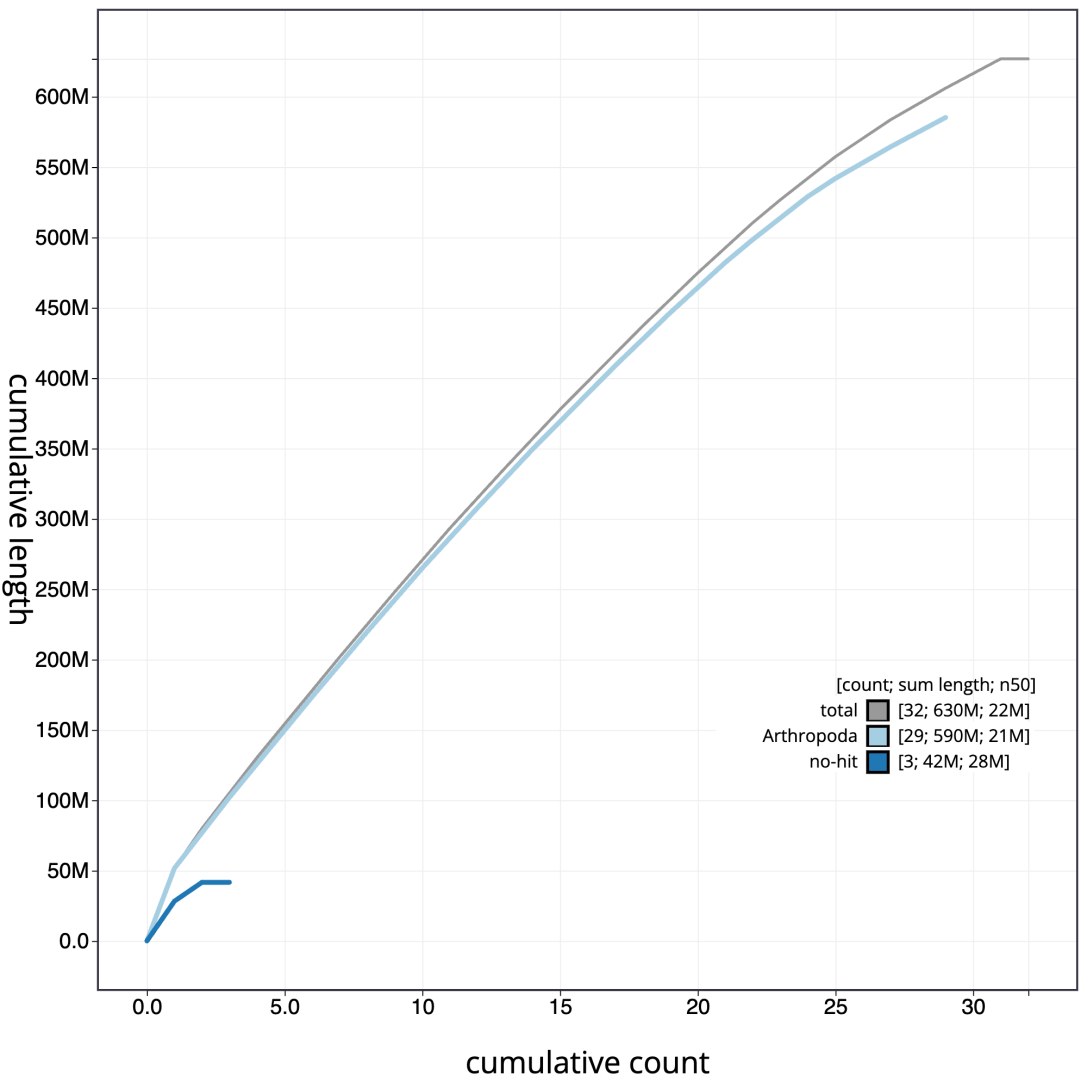
Genome assembly of *Archips crataeganus*, ilArcCraa1.1: BlobToolKit cumulative sequence plot. The grey line shows cumulative length for all scaffolds. Coloured lines show cumulative lengths of scaffolds assigned to each phylum using the buscogenes taxrule. An interactive version of this figure is available at https://blobtoolkit.genomehubs.org/view/Archips%20crataeganus/dataset/ilArcCraa1_1/cumulative.

**Figure 5.**
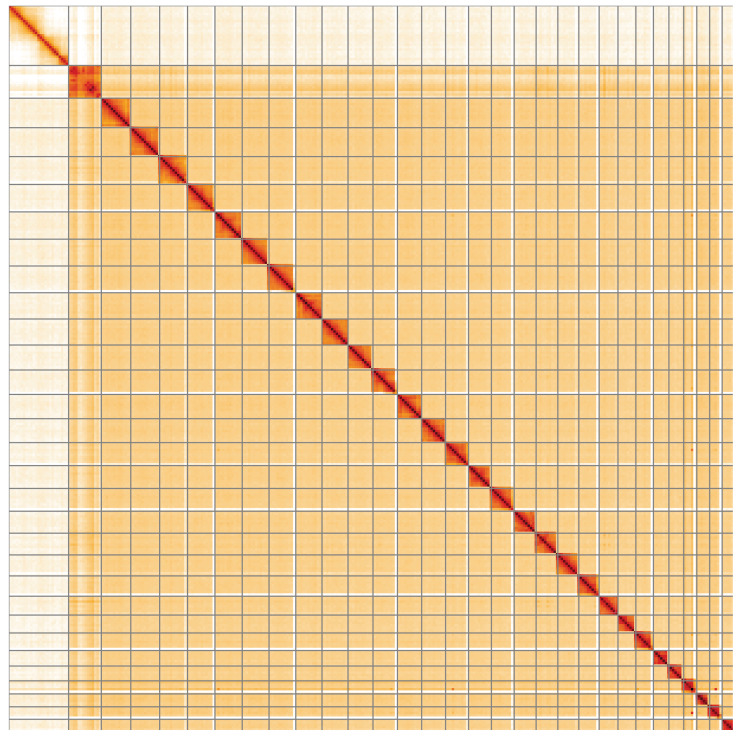
Genome assembly of *Archips crataeganus*, ilArcCraa1.1: Hi-C contact map of the ilArcCraa1.1 assembly, visualised using HiGlass. Chromosomes are shown in order of size from left to right and top to bottom. An interactive version of this figure may be viewed at https://genome-note-higlass.tol.sanger.ac.uk/l/?d=E3hPUivPSOK66eemQfiTVA.

The final assembly has a total length of 626.9 Mb in 31 sequence scaffolds with a scaffold N50 of 21.6 Mb ( Table 1). The snailplot in Figure 2 provides a summary of the assembly statistics, while the distribution of assembly scaffolds on GC proportion and coverage is shown in Figure 3. The cumulative assembly plot in Figure 4 shows curves for subsets of scaffolds assigned to different phyla. Most (99.99%) of the assembly sequence was assigned to 31 chromosomal-level scaffolds, representing 29 autosomes and the W and Z sex chromosomes. There is some uncertainty to the order and orientation of W chromosome contigs. Chromosome-scale scaffolds confirmed by the Hi-C data are named in order of size ( Figure 5; Table 2). While not fully phased, the assembly deposited is of one haplotype. Contigs corresponding to the second haplotype have also been deposited. The mitochondrial genome was also assembled and can be found as a contig within the multifasta file of the genome submission.

The estimated Quality Value (QV) of the final assembly is 66.3 with *k*-mer completeness of 100%, and the assembly has a BUSCO v5.3.2 completeness of 98.2% (single = 97.8%, duplicated = 0.4%), using the lepidoptera_odb10 reference set ( *n* = 5,286).

**Table 1.** Genome data for *Archips crataeganus*, ilArcCraa1.1.

Project accession data		
Assembly identifier	ilArcCraa1.1	
Species	*Archips crataeganus*	
Specimen	ilArcCraa1	
NCBI taxonomy ID	1857967	
BioProject	PRJEB56800	
BioSample ID	SAMEA10978952	
Isolate information	ilArcCraa1, female: whole organism (DNA sequencing and Hi-C data)	
Assembly metrics *		*Benchmark*
Consensus quality (QV)	66.3	*≥ 50*
*k*-mer completeness	100%	*≥ 95%*
BUSCO **	C:98.2%[S:97.8%,D:0.4%],F:0.4%,M:1.4%,n:5,286	*C ≥ 95%*
Percentage of assembly mapped to chromosomes	99.99%	*≥ 95%*
Sex chromosomes	W and Z chromosome	*localised homologous pairs*
Organelles	Mitochondrial genome assembled	*complete single alleles*
Raw data accessions		
PacificBiosciences SEQUEL II	ERR10395977	
Hi-C Illumina	ERR10395983	
Genome assembly		
Assembly accession	GCA_947859365.1	
*Accession of alternate haplotype*	GCA_947859345.1	
Span (Mb)	626.9	
Number of contigs	158	
Contig N50 length (Mb)	8.2	
Number of scaffolds	31	
Scaffold N50 length (Mb)	21.6	
Longest scaffold (Mb)	51.7	
Genome annotation		
Number of protein-coding genes	19,596	
Number of gene transcripts	19,756	

* Assembly metric benchmarks are adapted from column VGP-2020 of “Table 1: Proposed standards and metrics for defining genome assembly quality” from ( Rhie *et al.*, 2021). ** BUSCO scores based on the lepidoptera_odb10 BUSCO set using v5.3.2. C = complete [S = single copy, D = duplicated], F = fragmented, M = missing, n = number of orthologues in comparison. A full set of BUSCO scores is available at https://blobtoolkit.genomehubs.org/view/Archips%20crataeganus/dataset/ilArcCraa1_1/busco.

**Table 2.** Chromosomal pseudomolecules in the genome assembly of *Archips crataeganus*, ilArcCraa1.

INSDC accession	Chromosome	Length (Mb)	GC%
OX402047.1	1	25.48	38.5
OX402048.1	2	24.98	38.0
OX402049.1	3	24.11	38.0
OX402050.1	4	23.69	38.0
OX402051.1	5	23.6	38.5
OX402052.1	6	23.18	38.5
OX402053.1	7	23.12	38.0
OX402054.1	8	22.7	38.0
OX402055.1	9	22.48	38.0
OX402056.1	10	21.61	38.0
OX402057.1	11	21.13	38.0
OX402058.1	12	20.96	38.5
OX402059.1	13	20.67	38.5
OX402060.1	14	19.87	38.5
OX402061.1	15	19.74	38.5
OX402062.1	16	19.69	38.5
OX402063.1	17	18.98	38.5
OX402064.1	18	18.77	38.5
OX402065.1	19	18.15	38.5
OX402066.1	20	17.57	38.5
OX402067.1	21	16.29	39.0
OX402068.1	22	15.48	39.0
OX402069.1	23	15.17	38.5
OX402070.1	24	13.4	39.0
OX402071.1	25	12.8	39.0
OX402072.1	26	11.32	39.5
OX402073.1	27	11.12	39.5
OX402074.1	28	10.78	39.0
OX402075.1	29	10.08	39.0
OX402046.1	W	28.27	39.0
OX402045.1	Z	51.69	38.0
OX402076.1	MT	0.02	18.0

Metadata for specimens, barcode results, spectra estimates, sequencing runs, contaminants and pre-curation assembly statistics are given at https://links.tol.sanger.ac.uk/species/1857967.

## Genome annotation report

The *Archips crataeganus* genome assembly (GCA_947859365.1) was annotated using the Ensembl rapid annotation pipeline ( Table 1; https://rapid.ensembl.org/Archips_crataeganus_GCA_947859365.1/Info/Index). The resulting annotation includes 19,756 transcribed mRNAs from 19,596 protein-coding genes.

## Methods

### Sample acquisition and nucleic acid extraction

A female *Archips crataeganus* (specimen ID Ox001685, ToLID ilArcCraa1) was collected from Wytham Woods, Oxfordshire (biological vice-county Berkshire), UK (latitude 51.77, longitude –1.34) on 2021-07-17 using a light trap. The specimen was collected and identified by Douglas Boyes (University of Oxford) and preserved on dry ice.

The workflow for high molecular weight (HMW) DNA extraction at the Wellcome Sanger Institute (WSI) includes a sequence of core procedures: sample preparation; sample homogenisation; DNA extraction; HMW DNA fragmentation; and fragmented DNA clean-up. The sample was prepared for DNA extraction at the WSI Tree of Life laboratory: the ilArcCraa1 sample was weighed and dissected on dry ice with tissue set aside for Hi-C sequencing ( https://dx.doi.org/10.17504/protocols.io.x54v9prmqg3e/v1). Tissue from the whole organism was disrupted using a Nippi Powermasher fitted with a BioMasher pestle ( https://dx.doi.org/10.17504/protocols.io.5qpvo3r19v4o/v1). DNA was extracted at the WSI Scientific Operations core using the Qiagen MagAttract HMW DNA kit, according to the manufacturer’s instructions.

Protocols developed in the Tree of Life laboratory are publicly available on protocols.io ( https://dx.doi.org/10.17504/protocols.io.8epv5xxy6g1b/v1).

### Sequencing

Pacific Biosciences HiFi circular consensus DNA sequencing libraries were constructed according to the manufacturers’ instructions. DNA sequencing was performed by the Scientific Operations core at the WSI on a Pacific Biosciences SEQUEL II instrument. Hi-C data were also generated from remaining tissue of ilArcCraa1 using the Arima2 kit and sequenced on the Illumina NovaSeq 6000 instrument.

### Genome assembly, curation and evaluation

Assembly was carried out with Hifiasm ( Cheng *et al.*, 2021) and haplotypic duplication was identified and removed with purge_dups ( Guan *et al.*, 2020). The assembly was then scaffolded with Hi-C data ( Rao *et al.*, 2014) using YaHS ( Zhou *et al.*, 2023). The assembly was checked for contamination and corrected as described previously ( Howe *et al.*, 2021). Manual curation was performed using HiGlass ( Kerpedjiev *et al.*, 2018) and Pretext ( Harry, 2022). The mitochondrial genome was assembled using MitoHiFi ( Uliano-Silva *et al.*, 2023), which runs MitoFinder ( Allio *et al.*, 2020) or MITOS ( Bernt *et al.*, 2013) and uses these annotations to select the final mitochondrial contig and to ensure the general quality of the sequence.

A Hi-C map for the final assembly was produced using bwa-mem2 ( Vasimuddin *et al.*, 2019) in the Cooler file format ( Abdennur & Mirny, 2020). To assess the assembly metrics, the *k*-mer completeness and QV consensus quality values were calculated in Merqury ( Rhie *et al.*, 2020). This work was done using Nextflow ( Di Tommaso *et al.*, 2017) DSL2 pipelines “sanger-tol/readmapping” ( Surana *et al.*, 2023a) and “sanger-tol/genomenote” ( Surana *et al.*, 2023b). The genome was analysed within the BlobToolKit environment ( Challis *et al.*, 2020) and BUSCO scores ( Manni *et al.*, 2021; Simão *et al.*, 2015) were calculated.

Table 3 contains a list of relevant software tool versions and sources.

**Table 3.** Software tools: versions and sources.

Software tool	Version	Source
BlobToolKit	4.2.1	https://github.com/blobtoolkit/blobtoolkit
BUSCO	5.3.2	https://gitlab.com/ezlab/busco
Hifiasm	0.16.1-r375	https://github.com/chhylp123/hifiasm
HiGlass	1.11.6	https://github.com/higlass/higlass
Merqury	MerquryFK	https://github.com/thegenemyers/MERQURY.FK
MitoHiFi	2	https://github.com/marcelauliano/MitoHiFi
PretextView	0.2	https://github.com/wtsi-hpag/PretextView
purge_dups	1.2.3	https://github.com/dfguan/purge_dups
sanger-tol/genomenote	v1.0	https://github.com/sanger-tol/genomenote
sanger-tol/readmapping	1.1.0	https://github.com/sanger-tol/readmapping/tree/1.1.0
YaHS	yahs-1.1.91eebc2	https://github.com/c-zhou/yahs

### Genome annotation

The BRAKER2 pipeline ( Brůna *et al.*, 2021) was used in the default protein mode to generate annotation for the *Archips crataeganus* assembly (GCA_947859365.1) in Ensembl Rapid Release.

### Wellcome Sanger Institute – Legal and Governance

The materials that have contributed to this genome note have been supplied by a Darwin Tree of Life Partner. The submission of materials by a Darwin Tree of Life Partner is subject to the **‘Darwin Tree of Life Project Sampling Code of Practice’**, which can be found in full on the Darwin Tree of Life website here. By agreeing with and signing up to the Sampling Code of Practice, the Darwin Tree of Life Partner agrees they will meet the legal and ethical requirements and standards set out within this document in respect of all samples acquired for, and supplied to, the Darwin Tree of Life Project.

Further, the Wellcome Sanger Institute employs a process whereby due diligence is carried out proportionate to the nature of the materials themselves, and the circumstances under which they have been/are to be collected and provided for use. The purpose of this is to address and mitigate any potential legal and/or ethical implications of receipt and use of the materials as part of the research project, and to ensure that in doing so we align with best practice wherever possible. The overarching areas of consideration are:

•  Ethical review of provenance and sourcing of the material

•  Legality of collection, transfer and use (national and international) 

Each transfer of samples is further undertaken according to a Research Collaboration Agreement or Material Transfer Agreement entered into by the Darwin Tree of Life Partner, Genome Research Limited (operating as the Wellcome Sanger Institute), and in some circumstances other Darwin Tree of Life collaborators.

## Data Availability

European Nucleotide Archive: *Archips crataeganus* (brown oak tortrix). Accession number PRJEB56800; https://identifiers.org/ena.embl/PRJEB56800 ( Wellcome Sanger Institute, 2023). The genome sequence is released openly for reuse. The *Archips crataeganus* genome sequencing initiative is part of the Darwin Tree of Life (DToL) project. All raw sequence data and the assembly have been deposited in INSDC databases. Raw data and assembly accession identifiers are reported in Table 1.

## Author information

Members of the University of Oxford and Wytham Woods Genome Acquisition Lab are listed here: https://doi.org/10.5281/zenodo.7125292.

Members of the Darwin Tree of Life Barcoding collective are listed here: https://doi.org/10.5281/zenodo.4893703.

Members of the Wellcome Sanger Institute Tree of Life programme are listed here: https://doi.org/10.5281/zenodo.4783585.

Members of Wellcome Sanger Institute Scientific Operations: Sequencing Operations collective are listed here: https://doi.org/10.5281/zenodo.10043364.

Members of the Tree of Life Core Informatics collective are listed here: https://doi.org/10.5281/zenodo.5013541.

Members of the Darwin Tree of Life Consortium are listed here: https://doi.org/10.5281/zenodo.4783558.
