# Supplementary material for: The genome sequence of the Brown Oak Tortrix, Archips crataeganus (Hübner, 1796)
Source: Wellcome Open Res. 2024 Jan 8;9:9. [Version 1] doi: 10.12688/wellcomeopenres.20413.1 (PMC10980857; doi:10.12688/wellcomeopenres.20413.1)
Supplement: Peer review history

# Reviewer report: Reviewer response for version 1

**DOI:** 10.21956/wellcomeopenres.22601.r75953

**Published:** 2024-04-15

**Authors:** Andrew Mongue

**Affiliations:** 1 University of Florida, Gainesville, Florida, USA

Once again, another well assembled lepidopteran genome. I am very excited about the increasing number of W chromosomes captured by the DTOL project and commend the authors for their work assembling such messy chromosomes. 

 I am somewhat confused about the gene annotation side of this project however. The manuscript mentions a BRAKER2 annotation, but provides very little detail on how this was approached (e.g. was the assembly repeatmasked first?). Moreover, when checking the available data, it looks like RNA sequencing was generated (see: https://tolqc.cog.sanger.ac.uk/darwin/insects/Archips_crataeganus/) but it's unclear to me whether or not these RNA data were used in the annotation. 

 I recently reviewed another genome note with essentially the same lack of clarity around the annotation, so I hope this and future genome notes provide more detail on this front to make the most of the resources generated.

Are sufficient details of methods and materials provided to allow replication by others?

Partly

Is the rationale for creating the dataset(s) clearly described?

Yes

Are the datasets clearly presented in a useable and accessible format?

Partly

Are the protocols appropriate and is the work technically sound?

Yes

Reviewer Expertise:

genome assembly and annotation, Lepidoptera, population genetics, sex chromosomes

I confirm that I have read this submission and believe that I have an appropriate level of expertise to confirm that it is of an acceptable scientific standard, however I have significant reservations, as outlined above.

# Reviewer report: Reviewer response for version 1

**DOI:** 10.21956/wellcomeopenres.22601.r75954

**Published:** 2024-04-15

**Authors:** Sean T S Law

**Affiliations:** 1 The Chinese University of Hong Kong, Hong Kong, Hong Kong

This article presents the genome of a female Brown Oak Tortrix, *Archips crataeganus*. The genome is ~627 Mb in size with scaffolds anchored into 31 pseudomolecules, including the Z and W sex chromosomes as the first two largest chromosomes. The high completeness and continuity of the genome assembly were supported by BUSCO score of 98.2% and N50 statistics. In summary, the genome assembly of *A. crataeganus* is of high-quality.

Are sufficient details of methods and materials provided to allow replication by others?

Yes

Is the rationale for creating the dataset(s) clearly described?

Yes

Are the datasets clearly presented in a useable and accessible format?

Yes

Are the protocols appropriate and is the work technically sound?

Yes

Reviewer Expertise:

Comparative genomics

I confirm that I have read this submission and believe that I have an appropriate level of expertise to confirm that it is of an acceptable scientific standard.

# Reviewer report: Reviewer response for version 1

**DOI:** 10.21956/wellcomeopenres.22601.r75962

**Published:** 2024-03-29

**Authors:** Annabel Whibley

**Affiliations:** 1 The University of Auckland, Auckland, Auckland, New Zealand; 2 Bragato Research Institute, Blenheim, New Zealand

The authors report a chromosomally-complete assembly of the Brown Oak Tortrix moth using PacBio HiFi reads (run on the Sequel II platform) and an Illumina Hi-C dataset. The resulting assembly and methods are clearly presented, using DToL protocols and pipelines. The assembly statistics are impressive and include 100% k-mer completeness. The HiC map looks phenomenal and the quality of this assembly will make it a wonderful resource for the community.

 I have just very minor comments:

*Archips *could be abbreviated throughout the manuscript after its first introduction.

 And while the protocols and reporting in these assembly notes are very succinctly and tightly composed, at times they are perhaps too sparse. For example, I would like to see the kmer size used in Merqury noted (the default in FastK upstream of the  MerquryFK  is 40, whereas the original Merqury Meryl implementation is typically used with 19/21-mers when assessing the accessory script is provided; k-mer choice has an impact on the reported completeness).

 In previous reports I have noted that the HiC reporting states that YAHS is used to scaffold without describing the alignment and processing steps prior to invoking YAHS, when the YAHS Github documentation notes that there are multiple routes to generating the inputs.

Are sufficient details of methods and materials provided to allow replication by others?

Yes

Is the rationale for creating the dataset(s) clearly described?

Yes

Are the datasets clearly presented in a useable and accessible format?

Yes

Are the protocols appropriate and is the work technically sound?

Yes

Reviewer Expertise:

Genomics; Evolution; Bioinformatics

I confirm that I have read this submission and believe that I have an appropriate level of expertise to confirm that it is of an acceptable scientific standard.

# Reviewer report: Reviewer response for version 1

**DOI:** 10.21956/wellcomeopenres.22601.r72743

**Published:** 2024-02-02

**Authors:** Fahad Alqahtani

**Affiliations:** 1 King Abdulaziz City for Science and Technology, Riyadh, Saudi Arabia

The authors state that they were successfully reconstructing chromosomal level genome from a female *Archips crataeganus*. The genome assembly was reconstructed using two technologies: Pacific Biosciences SEQUEL II and Hi-C Illumina. The genome is 626.9 Mb in size and contains 19,596 proteins coding genes. The genome completeness was assessed using BUSCO analysis, which showed that 98.2% of common genes were completely present. The analysis utilized the lepidoptera-odb10 lineage dataset, which contains 5,286 genes.

 However, a few minor points require attention: 

- I would suggest including a phylogenetic analysis of the mitochondrial genome, as it has the potential to enhance the impact of the work. 
- Regrading the mitochondrial genome annotation, the MitoHiFi tool, which was utilized with either MITOS or Mitofinder. Which of these tools was used for the genome's annotation? Furthermore, if Mitofinder was used, known for utilizing closely related species to guide annotation, I recommend including this species in the methods section.
- Lastly, the tool name 'Pretext' in the Genome Assembly section should be corrected to 'PretextView'."

Are sufficient details of methods and materials provided to allow replication by others?

Yes

Is the rationale for creating the dataset(s) clearly described?

Yes

Are the datasets clearly presented in a useable and accessible format?

Yes

Are the protocols appropriate and is the work technically sound?

Yes

Reviewer Expertise:

Bioinformatics

I confirm that I have read this submission and believe that I have an appropriate level of expertise to confirm that it is of an acceptable scientific standard.
